# Don't tell on me: Experimental evidence of asymmetric information in transnational households*

**DOI:** 10.1016/j.jdeveco.2014.11.001

**Published:** 2015-03-01

**Authors:** Kate Ambler

**Affiliations:** Markets, Trade, and Institutions Division, International Food Policy Research Institute, 2033 K St, NW, Washington, DC 20006

**Keywords:** Remittances, Intra-household allocation, Information asymmetries, Transnational households

## Abstract

Although most theoretical models of household decision making assume perfect information, empirical studies suggest that information asymmetries can have large impacts on resource allocation. I demonstrate the importance of these asymmetries in transnational households, where physical distance between family members can make information barriers especially acute. I implement an experiment among migrants in Washington, DC, and their families in El Salvador that examines how information asymmetries can have strategic and inadvertent impacts on remittance decisions. Migrants make an incentivized decision over how much of a cash windfall to remit, and recipients decide how they will spend a remittance. Migrants strategically send home less when their choice is not revealed to recipients. Recipients make spending choices closer to migrants' preferences when the migrants' preferences are shared, regardless of whether or not the spending choices are revealed to the migrants, suggesting that recipients' choices are inadvertently affected by imperfect information.

## 1. Introduction and motivation

Although the implications of asymmetric information have been well documented in the study of economic institutions such as labor, credit, and insurance markets, theoretical models of intra-household resource allocation have largely assumed perfect information (Chiappori, 1988, 1992; Lundberg and Pollack, 1993; Manser and Brown, 1980; McElroy and Horney, 1981).^1^ Despite this, a growing body of empirical literature has shown that information asymmetries do exist in households and, further, that household members take strategic advantage of these asymmetries to alter the allocation of resources in the household. For example, Ashraf (2009) shows that in the Philippines, men hide income from their wives when that decision is private and divert income to committed consumption when their decision is public. Only when spouses communicate about their choices before they make them do men choose to share the income with their wives. This paper brings the study of how information asymmetries affect intra-household resource allocation to a different setting: transnational households, defined as households composed of international migrants and their family members in the home country, in this case El Salvador. Using experimental methods, I examine the effects of a set of information imperfections on remittance decisions in a matched sample of migrants from El Salvador and their family members at home.

I address two types of information asymmetries that may affect decisions about the sending and spending of remittances. The first are asymmetries that can lead to *strategic* behavior, meaning that migrants and recipients recognize that the asymmetry exists and use it for their benefit. The specific asymmetries considered here are the limited abilities of remittance recipients to observe migrant income and of migrants to observe recipient spending. The second type are those that can have *inadvertent* impacts, defined as asymmetries that unintentionally affect decisions. These asymmetries are represented here as communication barriers that result in recipients having an incomplete understanding of migrant preferences for how the remittances they send should be spent. Communication barriers should be interpreted broadly as any obstacle—social, financial, or logistical—to understanding these preferences.

The context of transnational households is significant because migrants and their family members are making financial decisions in a situation where information asymmetries are especially acute due to the distance separating family members. A number of studies have documented the existence of these asymmetries in households with migrants. De Laat (2014) shows that domestic migrants in Kenya spend resources on costly monitoring of their wives. Chen (2013) finds that in China, wives with migrant husbands exhibit non-cooperative behavior more often for activities that are more difficult to monitor. McKenzie et al. (2013) find that potential Tongan migrants underestimate earnings in New Zealand, a fact the authors partly attribute to underreporting of earnings by migrants. Among Indian migrants in Qatar, Seshan and Yang (2014) find evidence that migrants underestimate how much their wives at home are saving, and Seshan and Zubrickas (2014) show that the underreporting of husband's income abroad by wives is correlated with lower remittances. However, the empirical analysis in these papers is largely observational. This is the first study to causally examine how information asymmetries directly affect behavior, specifically decisions about the sending and spending of remittances.

The importance of understanding how information asymmetries affect decisions in transnational households is heightened by the fact that migrants and their family members are financially linked through the sending of remittances. In 2010, aggregate remittances to the developing world were US$332 billion, and in El Salvador remittances received were 16% of GDP (Ratha and Silwal, 2012). Additionally, the receipt of remittances has been shown to have positive impacts on a variety of measures of well-being (Adams and Cuecuecha, 2010; Adams and Page, 2005; Cox Edwards and Ureta, 2003; Woodruff and Zenteno, 2007; Yang, 2008; Yang and Martinez, 2005). Given this importance of remittances in developing countries, a more complete understanding of how remittance decisions are made is crucial for policymakers who are hoping to maximize their economic impact.

A growing experimental literature related to remittances and information has focused on the strategic efforts of migrants to control remittance spending. Specifically, several field experiments have examined the effects of offering migrants varying degrees of control over remittances. The idea behind these experiments is that offering control to migrants will mitigate a moral hazard problem in how recipients spend remittances. Studies have found mixed results. Ashraf et al. (forthcoming) show that savings levels in bank accounts in El Salvador increase when migrants are given greater control over these accounts. Chin et al. (2011) find that the impacts of an experiment that offered migrants assistance in opening bank accounts in the United States are concentrated among migrants who report having no control over how their remittances are spent. However, in a lab experiment, Torero and Viceisza (2013) do not find that Salvadoran migrants send more when they are able to control how remittances are spent, and attribute this to the fact that the control offered by their experiment was too limiting. Additionally, Ambler et al. (forthcoming) find no unsubsidized demand for a product that directs remittances to education and De Arcangelis et al. (2014) show evidence that simply labeling remittances for education is as effective in increasing remittances as providing a hard commitment device.

A limitation of the papers described above is that they do not consider that information problems might run in both directions. One of the principal contributions of this paper is therefore that it examines the strategic impacts of information asymmetries on *both* sides of the migrant–recipient relationship. The observational studies documenting information asymmetries in migrant households have also largely focused on migrant monitoring of recipient behavior (Chen, 2013; de Laat, 2014) and none examine the decisions of both migrants and recipients.

This study also fits into the literature that describes information asymmetries and strategic behavior in families that live in closer proximity. In addition to the previously described Ashraf (2009) study in the Philippines, Schaner (forthcoming) finds that spouses are more likely to choose to save in individual (as opposed to joint) savings accounts when they are not well informed about each other's finances. Also related, Jakiela and Ozier (2012) find that women in Kenya sacrifice investment returns to keep income secret from family members outside their household and avoid the pressure to share that income. Ashraf et al. (2014), Castilla and Walker (2013), and Hoel (2014) also find evidence of strategic effects of information asymmetries between spouses.

An additional contribution of this paper is that it also addresses the inadvertent impacts of information asymmetries, which have been studied much less. In a field experiment with migrants in Ireland, Batista and Narciso (2013) find that providing migrants with free phone calls to their home country increases both communication with family members and remittances. However, although this experiment lowers communication costs, it cannot identify whether the information asymmetry alleviated was strategic or inadvertent. For example, increased communication may have given migrants better information about recipient needs, but it also may have reduced the ability of migrants to strategically underreport their income. Similarly, the communication treatment in Ashraf (2009) allows spouses to discuss their decisions, and therefore cannot distinguish a strategic bargaining effect from an alleviation of an inadvertent information barrier.

The experiments in this paper explicitly test for both strategic and inadvertent responses to information asymmetries. They were designed to mimic real-life decisions about remittances made by migrants and their family members, and by randomly assigning treatments, I am able to causally identify the impacts of the informational conditions being tested. The first experiment was conducted among Salvadoran migrants recruited in the Washington, DC, area.^2^ The migrants were asked how much of a possible $600 lottery prize they wished to keep and how much they wished to send to a family member in El Salvador. Participants had the chance to win the allocation that they chose. To test whether migrants strategically react to changes in the observability of their income, they were randomly allocated into two treatment groups: those whose decisions were revealed to their families back home (and the migrants were told their decisions would be revealed), and those whose decisions were not revealed to their families at home (and, similarly, the migrants were told their decisions would remain private). Although the choice was described to the migrant as a remittance decision, it should be noted that the migrant experiment is essentially a version of the classic dictator game where one player allocates a sum of money between him or herself and a recipient.

The migrants' family members in El Salvador (referred to throughout the paper as “recipients”) then participated in a second experiment. They made a decision about how to spend a possible $300 lottery prize. This prize was referred to as a remittance and recipients were told that they were eligible for this remittance because their migrant family member was participating in the study. To test for strategic reactions to the observability of their spending choices, as in the migrant experiment half of the recipients were told that their choice would not be revealed to the migrant, and the other half were told that their choice would be revealed. In a second cross-randomized treatment addressing the inadvertent effects of communication barriers, half of the recipients were informed of the migrant's preferences for how the money should be spent, and the others were not. These preferences were collected from migrants during the migrant survey, following the conclusion of the migrant experiment described above. In order to avoid the migrant experiment influencing the results of the recipient experiment, the information revelations about choices made in the migrant experiment and the awarding of all prizes were conducted after the conclusion of both experiments.

I find that migrants remit $20 more on average out of the possible $600 lottery prize (an increase of 5% over the control group mean of $440 sent) when their decisions are revealed. I additionally report suggestive evidence that this effect is concentrated among migrants whose baseline characteristics indicate that they may be likely to react strategically to the opportunity to hide income from their family members. These characteristics include time spent in the United States, relationship between the recipient and the migrant, whether the migrant has a child 22 or under in El Salvador, frequency of communication between the migrant and the recipient, and remittances sent by the migrant to the recipient. There is no corresponding evidence of strategic behavior in the recipient experiment. Therefore, despite the focus on migrant monitoring of recipient behavior in the existing literature, I find that migrants, but not recipients, react strategically to whether or not their choices will be monitored. In the second recipient treatment reducing communication costs by revealing migrant preferences to recipients does have an impact, resulting in a 10% reduction in the difference between migrant preferences and recipient choices. The effect is present even when recipient choices are not revealed to migrants. This result demonstrates that information barriers can have inadvertent impacts on remittance decisions.

In addition to its principal contributions to the literature on information issues in migrant families, this study also builds on the existing household literature more generally. For example, most papers focus on only a single choice in the resource allocation process, and the present experiment considers how information asymmetries can affect a sequence of two different decisions made by families about economic resources. Additionally, this study documents that information asymmetries can be important outside of the husband–wife pair, which has been the context of most previous experimental work in this area.^3^ People in developing countries often transfer resources within extended families, and therefore decisions about resource allocation are likely to involve people beyond just the husband and wife. These results show that information asymmetries can have important impacts in extended families.

Because the design of the migrant experiment is essentially a modified dictator game it is also important to understand how this study differs from the extensive existing literature on dictator games. Dictator games are generally used as a test for altruistic behavior and are played anonymously, with neither the dictator nor the recipient knowing the identity of the other. Results generally show that dictators deviate from pure self-interest and allocate around 20% of the available funds to the recipient (Camerer, 2003; Cardenas and Carpenter, 2008). Studies have shown that both making the identity of the recipient known to the dictator and revealing the dictator choices to the recipient substantially raise giving (Bohnet and Frey, 1999; Leider et al., 2009; Ligon and Schechter, 2012).

The migrant experiment can be thought of as a dictator game with known recipients that studies the impact of revealing dictator choices similar to studies such as Leider et al. (2009) and Ligon and Schechter (2012). However, the literature has also shown that the culture of the participants and the framing of the game can have large effects on choices made (Cardenas and Carpenter, 2008; Henrich et al., 2005; Levitt and List, 2007). Therefore, given the study population and the framing of the game as a remittance decision, the results cannot necessarily be predicted based on previous studies. Additionally, a unique element of this experiment is that dictator games are not normally run among people who regularly transfer large sums of money to each other. Therefore, this study provides a setting in which the results of the game can be more closely linked to behavior outside the experimental setting.^4^

The paper proceeds as follows: Section 2 describes a simple conceptual framework. Section 3 explains the experimental design. Section 4 describes the data and the empirical strategy. Section 5 presents the results, and Section 6 concludes.

## 2. Framework

### 2.1. Migrant decision

In this section I describe a simple economic framework that motivates the experimental design. The migrant's decision about how much to send home in remittances is driven by two factors: altruism towards the recipient and additional costs that vary with the remittance amount. These costs are broadly construed and meant to encompass most other motivations for sending remittances previously considered in the literature.^5^ The idea is that migrants are compelled to send more in remittances than they would choose to if simply motivated by altruism because they want to avoid incurring these costs. One example of such a cost is social sanctions against the migrant: many migrants come from areas with strong social norms regarding remittances and particularly for migrants who wish to return home one day, a damaged reputation could be quite costly. Empirical support for social norms and recipient pressure driving remittances includes De Weerdt and Hirvonen (2013) and De Weerdt et al. (2014). Another example is substandard care for people (children or elderly relatives) or possessions (land, livestock, or new investments) left by the migrant in the care of his family. Migrants who fail to send home as much money as their families expect may also damage their relationships with their families. Conversely, migrants may also incur a benefit from appearing fair or altruistic towards their family members (Bénabou and Tirole, 2006) which could lead them to send larger amounts.^6^

The expected remittance payment depends on the recipient's perception of the migrant's incomes. For example, if migrants are earning more, social norms may dictate that larger amounts should be sent home. Similarly, recipients will perceive migrants to be more altruistic the larger the proportion of their overall income that is remitted. The extent to which recipients have knowledge of migrants' true incomes will vary. In some circumstances recipients may have independent information about how much the migrant has earned,^7^ but in many cases they must rely only on what the migrant has told them. Therefore, migrants for whom these costs are important can strategically take advantage of situations where recipients cannot observe their true income to underreport their earnings and send less home.

### 2.2. Recipient decision

The decision made by recipients about how to spend the remittances that they receive is considered in a parallel manner to the migrant sending decision. Recipients choose the extent to which they follow the migrants' preferences for how remittances should be spent. Their choice is motivated by altruism (in this case simply the extent to which recipients want to follow migrant preferences) and other costs related to the recipient's decision. For example, failure to comply with migrant preferences could lead to a reduction in remittance funds in the future. As with the migrant decision, recipients can therefore strategically take advantage of circumstances when their spending is unobservable by spending less money according to migrant preferences.

The recipient decision may additionally be complicated by barriers to communication that result in confusion over what the migrant's preferences actually are. I will refer to these barriers as *communication* costs, but the concept is broader than the cost of a telephone call. With distance, specificity about preferences may become difficult, migrants may feel uncomfortable expressing what they want, and recipients may sometimes have to make decisions without time to directly consult with migrants. Family members may also incorrectly assume that they know what the migrant would prefer. If the recipient does not know the migrant's preferences, the preferences will not be followed regardless of the recipient's intentions.

## 3. Experimental design

Given that information asymmetries in transnational families are difficult to measure and may be correlated with a number of unobserved characteristics, I implement a randomized experiment to test for their strategic and inadvertent impacts. This experiment is conducted within the context of survey work for a separate field experiment on remittances and education among Salvadoran migrants in Washington, DC, and their families in El Salvador (Ambler et al., forthcoming). The details of the companion experiment are described in Appendix A. This data collection exercise involves surveys with matched pairs of migrants and family members, allowing me to investigate the preferences and choices of both. In the experiment, I randomly vary (1) whether migrant income and recipient spending are observed and (2) the size of communication costs, allowing me to identify the causal impacts of both of these factors on migrant and recipient remittance behavior. The first variation tests for strategic reactions to information asymmetries, while the second looks for inadvertent impacts.

Migrants were recruited in the Washington, DC, metro area at the two area locations of the Salvadoran consulate and were interviewed while they were waiting for consular services. The migrant survey was conducted between late September 2011 and late February 2012. Surveyors in the consulate approached migrants and invited them to participate. Because the focus of the companion experiment was remittances and education, participants were required to name a high school or college-aged relative or acquaintance in El Salvador. Those who qualified and agreed to participate were administered a baseline survey. The migrant experiment described in this paper was conducted at the end of the survey. Following the experiment, the migrants responded to an additional question to be used in the recipient experiment.^8^

**Fig. 1:**
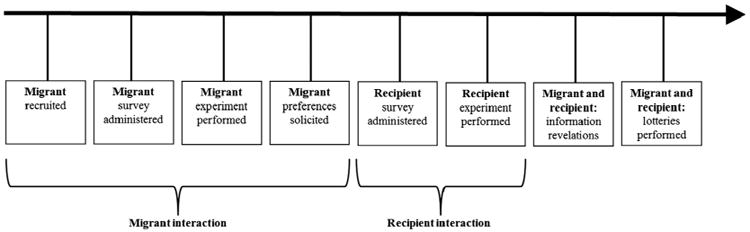
Project timeline.

During the survey migrants identified a high school or college-aged student in El Salvador. Interviews were subsequently conducted with the student or a household member. If the student was 18 years of age or older, the student was to be interviewed; and if under 18, a guardian was identified to be interviewed. If the indicated person was not available, an alternative adult in the household was interviewed. The El Salvador survey was conducted by phone in the days following the migrant survey in the United States; the median number of days between surveys was eight. The El Salvador surveys concluded in mid-March 2012, two weeks after fieldwork ended in the United States. The experiment in the El Salvador survey was also at the end of the survey. Fig. 1 describes the phases of the project in the order that they occurred for each pair of participants.

The randomization in this study was performed at the participant level. Surveys were pre-assigned treatment status, and randomization for all treatments was stratified within groups of 16 surveys and by the treatment offered in the companion experiment. The recipient treatments were additionally stratified by the migrant treatment.

### 3.1. Migrant experiment

The migrant experiment consisted of an incentivized remittance-sending decision. Migrants were told that they were being given the chance to win a $600 lottery and would have to decide how much of the prize to keep for themselves and how much to remit to their family member in El Salvador. Migrants could split the $600 as they wished but were restricted to using $100 intervals for simplicity. The prize was awarded through a lottery and two prizes were given out. If asked, surveyors told migrants the number of prizes and the date of the drawing.^9^ More than half the migrants who participated in this study report earning less than $400 a week. Consequently, $600 represents a significant increase in monthly income. The question text can be found in Appendix B.

**Fig. 2:**
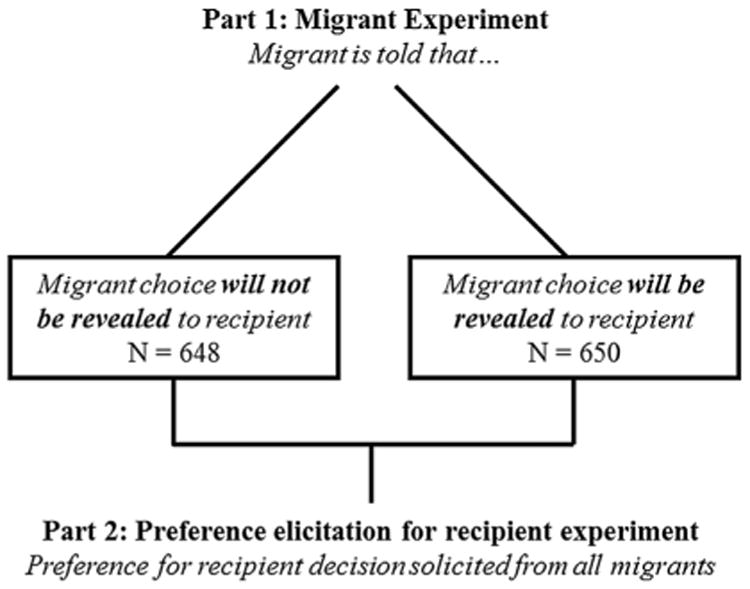
Migrant experiment.

Migrants were randomly allocated into two groups: those who were told that their choice would be revealed to their family member, and those who were told that their choice would not be revealed. For those in the choice revealed treatment group, all recipients were to be informed about the migrant's choice, regardless of whether or not they won the lottery. Recipients in the choice not revealed group were told nothing about the migrant experiment unless the migrant won the lottery and had elected to send the recipient some or all of the $600. The family member referred to in the question was the person to be surveyed in El Salvador. A description of the treatments is presented in Fig. 2. Because the treatment varies the ability of the recipient to monitor the migrant's actions, I refer to this treatment as the migrant monitoring treatment.

This experiment exogenously varies the probability that recipients will observe migrant income and measures the extent to which migrants strategically take advantage of asymmetric information. The framework described in Section 2 predicts that migrants whose choice is not revealed to the recipient should send less than those whose choice is revealed.

Because the prize was awarded through a lottery, participants were not guaranteed any winnings and the expected value of the prize for each participant was quite low.^10^ As the goal of the experiments was to emulate real life remittance decisions it was necessary to use money amounts that were close in magnitude to the sums participants would be considering when making these real life decisions. Given the average size of remittance payments in this population and budgetary restrictions, the lottery method was the only feasible method of conducting the study. This method has been used in many prior studies such as Armantier (2006), Murnighan and Saxon (1998), Ockenfels and Warner (2012), and Straub and Murnighan (1995). It has also been used specifically in studies that modeled remittances (De Arcangelis et al., 2014; Torero and Viceisza, 2013).,^11,12^

### 3.2. Recipient experiment

The recipient experiment consisted of an incentivized remittance spending decision. The respondents in the El Salvador phone survey were told that because their family member in the United States participated in the study, they now had the chance to win a lottery for a remittance worth $300. They had to decide what to spend the remittance on and were asked to split the $300 among four spending categories: restaurant meals, education, daily expenses, and health expenses. Recipient choices were limited to four categories for simplicity in the context of a phone survey. Immediately following the migrant experiment, during the US survey (which occurred prior to the recipient interview), all migrants were told about the lottery for recipients and asked what their preferences were for how the recipients would spend the money.^13^ This knowledge of the migrants' preferences will allow for a direct examination of how closely recipients adhere to these preferences.

**Fig. 3:**
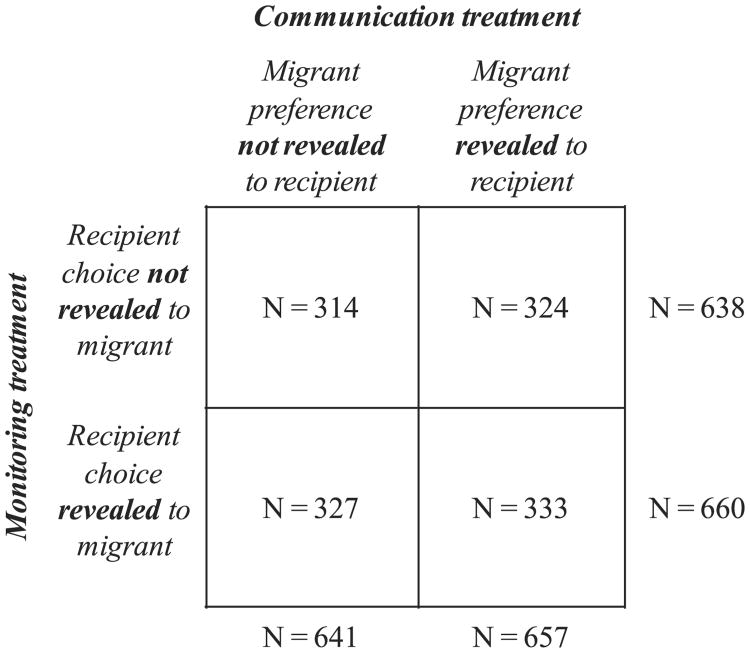
Recipient experiment.

Those recipients who won the lottery received exactly the allocations that they requested. Four prizes were awarded. If asked, surveyors told the recipients the number of prizes and the date of the drawing.^14^ Prizes were awarded in kind and respondents were held to the choices that they made. In practice this meant that project staff coordinated the delivery of the selected prize with each lottery winner on a case by case basis. Some choice was permitted within the categories the recipients had chosen but they could not request prizes outside of those categories. The median monthly remittance reported by participating migrants is $220, so a $300 remittance is a reasonable amount for many recipients. The question text can be found in Appendix B. Two separate, cross-randomized treatments were administered to recipients: the recipient monitoring treatment and the recipient communication treatment. They are depicted in Fig. 3.

### 3.3. Recipient monitoring treatment

The recipient monitoring treatment is parallel to the migrant monitoring treatment. Recipients were randomly allocated into two groups: those who were told that their choice would be revealed to the migrant, and those who were told that their choice would not be revealed to the migrant. Recipients knew that all migrants in the choice revealed treatment would be informed of their decision whether or not they won the lottery. Migrants in the choice not revealed group were told nothing. This treatment randomly varied the probability that recipient spending would be observed and measures the extent to which recipients strategically take advantage of this asymmetric information. Following the conceptual framework, the results of the experiment should show that recipients make choices closer to migrant preferences when those choices are revealed to the migrant.

### 3.4. Recipient communication treatment

As described above, during the US survey, migrants were told about the lottery for recipients and asked what their preferences were for how the recipients would spend the money. This information is utilized as part of this treatment. Recipients were randomly allocated into two groups: those for whom the migrant's preferences were revealed and those for whom the migrant's preferences were not revealed. Making these preferences clear is a proxy for improving communication, so the experiment exogenously improves communication about migrant preferences for expenditures. Revealing migrant preferences to the recipient should decrease the difference between the recipients' choices and the migrants' preferences when communication problems exist.

This treatment was designed as a test of whether or not communication costs can lead to inadvertent deviation from migrant preferences by the recipient. In order to make this argument convincing it is necessary to consider its interaction with the monitoring treatment. Specifically, if recipients are both uninformed and strategic, one would expect to only see effects for those recipients who are both told the migrants' preferences and told that their choices will be revealed. Conversely, a pure inadvertent effect can only be identified if there is an effect of the communication treatment for those recipients whose choices were *not* revealed to the migrant.

## 4. Data and estimation strategy

### 4.1. Data

Table 1 shows summary statistics from both the migrant and the recipient surveys: 1581 migrant surveys and 1298 recipient surveys were performed, a completion rate of 82% for the recipient surveys. For the migrant survey, summary statistics are shown for both the full sample and the sample with completed recipient surveys. Because no meaningful differences are evident between the two samples, I limit the analysis sample to the migrant–recipient pairs with completed El Salvador surveys. Results from the migrant experiment are similar across the two samples. The first row of Table 2 shows that attrition from the full sample of migrants to the estimation sample of migrant–recipient pairs with completed recipient surveys is not related to treatment. The breakdown of participants in the analysis sample into the different treatment groups can be seen in Fig. 2 (migrant experiment) and Fig. 3 (recipient experiment).

The migrants are half female with an average age of 38. The mean number of years in the United States is 11, so the migrants are largely established in the United States. 32% of migrants report having a son or daughter aged 22 or under in El Salvador, and 69% report communicating with the recipient household at least weekly. The sample is also low income; half of the migrants earn $400 a week or less. Because of the structure of the project, the interviewed recipients are either the student identified by the migrant (45%) or the student's guardian if the student is under 18 (40%). The remaining 15% of interviews were done with a different adult in the household if the student or guardian could not be reached. The recipient sample is heavily female (68%) because identified student guardians tend to be female.

Because migrants were screened into the study on the basis of having a young adult relative in El Salvador, it is possible that the respondents are not representative of the larger migrant community. This concern is heightened by the fact that many migrants were not eligible to participate because they did not know a student of the appropriate age. During recruitment, among the migrants approached 24% participated. Of those that did not participate, a considerable majority, 77%, did not know an eligible student in El Salvador.^15^ To address the external validity of the sample, in Appendix Table 1, I compare characteristics of the migrants from the survey (gender, age, time in the United States, household size, and education) tomigrants in the 2008–2010 American Community Survey (ACS) (Ruggles et al., 2010). I restrict the ACS sample to Salvadoran-born, non-US citizens aged 18 to 65 in the Washington, DC metro area. The study participants are quite similar to the ACS sample, suggesting that study participants are not overly different from the greater migrant population. However, while this comparison is useful it also does not cover a wide range of other characteristics. Therefore, the results of the experiments must be interpreted with the caveat that they were obtained using a selected sample. For example, given the screening criteria, this sample may have a greater interest in education than the average migrant in this area.

A related concern is that even if the migrants themselves are similar to the broader population of migrants in the area, the student age requirement results in migrants naming El Salvador households with which they are not typically in a remittance relationship. The evidence from the baseline survey refutes this. 85% of migrants have remitted to the recipient household in the past year and 78% of recipients are members of the migrant's home household in El Salvador. 64% send more to the recipient household than other households, indicating that the recipient household is the primary remittance recipient in the majority of cases. I also examine the relationship of the migrant to the recipient. A tabulation of these relationships is presented in Appendix Table 2. Almost all recipients are family members, and approximately 80% are parents, children, siblings, nieces or nephews, or cousins. The evidence is convincing that the sample is largely composed of migrant-recipient pairs involved in established remittance relationships, therefore making it an appropriate sample within which to study questions of information asymmetries.

Table 2 tests whether the treatment groups are balanced on observed characteristics from the baseline survey for both the migrant and the recipient experiment. The means by treatment group in the migrant monitoring treatment are presented in the first two columns, and the p-value of the test of whether or not those means are equal is in the third column. Overall, the treatment groups are well balanced: only 2 of 34 differences are significantly different from zero at the 10 percent level. Columns four through nine show the means by treatment group for the two recipient treatments and p-values for differences in those means. Only three of the 34 p-values for the recipient monitoring treatment and one for the recipient communication treatment are less than 0.10.

### 4.2. Estimation strategy: migrant experiment

The results of the migrant experiment can be analyzed by estimating the following regression using ordinary least squares:

(1) $${\text{Remit}}_{i}=\delta +{\alpha \text{ChoiceRevealed}}_{i}+\gamma_{i}+{ɛ}_{i}$$

where *Remit_i_* is the dependent variable indicating the amount that the migrant chose to send to the recipient. *ChoiceRevealed_i_* is the treatment indicator for the monitoring treatment, and it is equal to one when the migrant's choice is revealed to the recipient. The coefficient *α* is the average difference between how much migrants choose to send when their decisions are not revealed and when they are revealed. *γ_i_* are the stratification cell fixed effects. There are 111 survey group stratification cells in all regressions. I also include specifications with controls. The control variables include migrant age, gender, education, household size, years in the United States, remittances to recipient household, and other migrant background characteristics. *ε_i_* is the error term, which I adjust for heteroskedasticity.

### 4.3. Estimation strategy: recipient experiment

The recipient experiment was designed to understand the impacts of information asymmetries on the extent to which recipients follow migrant preferences for the expenditures of remittances. Because the migrant survey collected the migrant's preferences for the recipient's choices for all participants, it is possible to directly examine this parameter by comparing migrant preferences with recipient choices. I implement this by calculating the absolute value of the difference between the recipient's choice and the migrant's preference in each category. I also create a summary measure across the four categories by summing the difference variables and dividing by 2 to scale the total to 300. I refer to this as the total difference, and it is the primary dependent variable of interest. It is a measure of the number of dollars out of the $300 on which migrant and recipient choices match. For example, a total difference of 100 would mean the recipient's choices matched the migrant's preferences on $200 of the $300, but that they allocated the remaining $100 to different categories.^16^

An alternative estimation strategy would be to examine average recipient allocations to each spending category. However, in order to detect treatment effects using this strategy, migrant and recipient preferences would have to be different in the sample on average, for example if migrants overall preferred education while recipients preferred daily expenses. If migrants and recipients instead have approximately the same preferences on average, these averages can conceal substantial within-pair disagreement. Examination of only changes in average recipient allocations may then mask substantial changes in recipient behavior that will be evident when analyzing within-pair differences. Consequently, I focus on the pair-level differences, but discuss the impacts on average recipient allocations in Appendix C.

The recipient experiment can be analyzed by estimating the following regression:

(2) $${\text{Difference}}_{i}=\varphi +\beta_{1}{\text{ChoiceRevealed}}_{i}+\beta_{2}{\text{PreferenceRevealed}}_{i}+\beta_{3}{\text{ChoiceRevealed}}_{i}*{\text{PreferenceRevealed}}_{i}+\theta_{i}+\mu_{i}$$

where *Difference_i_* is the difference between migrant preferences and recipient choices in each of the four spending categories or the total difference. *ChoiceRevealed_i_* is the treatment indicator for the recipient monitoring treatment and is equal to one when the recipient's choice is revealed to the migrant. *PreferenceRevealed_i_* is the treatment indicator for the communication treatment and is equal to one when the migrant's preferences are revealed to the recipient before the recipient decides how to allocate the remittance funds. *ChoiceRevealed_i_* * *PreferenceRevealed_i_* is the interaction between the two treatments. If, as predicted, revealing the recipients' choices to the migrants and communicating the migrants' preferences to the recipients causes the recipients to make choices more similar to the migrants' preferences, even when the other treatment has not been applied, then the difference variable will be smaller in the choice revealed and preference revealed treatment groups, and *β*_1_ and *β*_2_ should be negative. If recipients react to being monitored more when the migrants' preferences are revealed (in other words they are both strategic and uninformed), then the interaction term will be important and *β*_3_ should be negative. *θ_i_* are the stratification cell fixed effects (survey group and migrant treatment). I also present a specification with control variables and use the same variables as in the migrant experiment as well as recipient gender, age, education, household size, and the number of days between the migrant and recipient surveys. *μ_i_* is the error term, which I adjust for heteroskedasticity.

## 5. Results

### 5.1. Migrant experiment

**Fig. 4:**
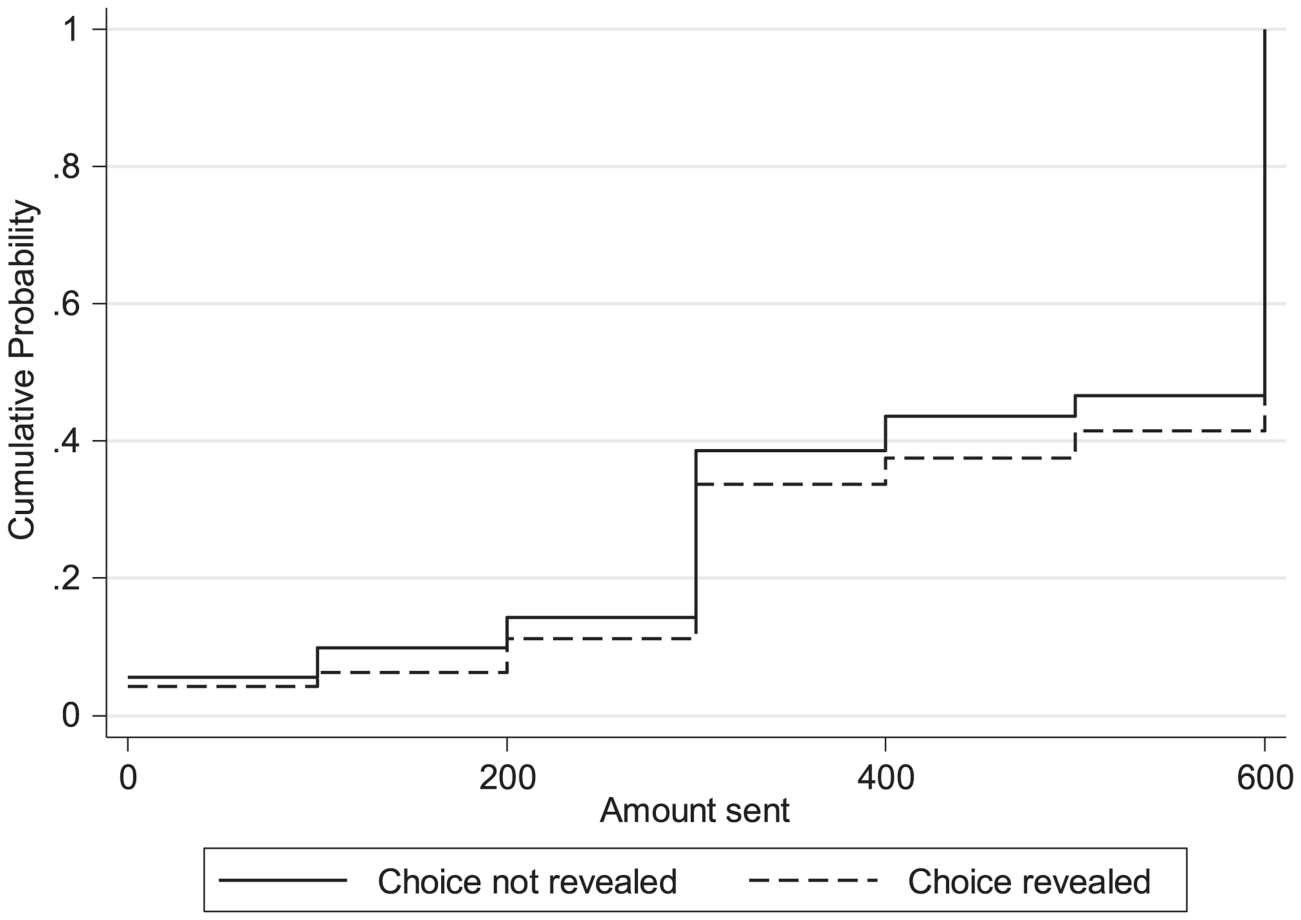
Cumulative distribution of amount sent by migrant by treatment group. Notes: Sample is observations with non-missing values for experiment questions and completed recipient survey. Choice not revealed: N = 648. Choice revealed: N = 650.

I first analyze the results of the migrant experiment in which migrants decide how much of a potential $600 lottery prize to send to the recipient and how much to keep. Fig. 4 shows the cumulative distribution of the amount sent by migrants, separately by treatment group. The first observation to be made from this figure is that the migrants send large amounts: more than half of the migrants in both treatment groups chose to send the entire potential prize. It should be noted that these amounts shared are much higher than normally found in dictator games, suggesting that the migrant context is an important one to study. Despite the fact that the two distributions follow the same basic shape, differences are evident. The percentage of migrants sending everything is smaller when choices are not revealed (53% versus 58%) and the percent of migrants choosing to send $400 or less is higher (44% versus 38%). It is also easy to see that the distribution of the choices in the choice revealed treatment group is always below the distribution of choices in the choice not revealed group; that is, the choice revealed distribution stochastically dominates the choice not revealed distribution.^17^

These results are formalized in Table 3, which presents the results of estimating regression Eq. (1). Column 1 is a simple regression of the dependent variable on treatment status, and column 2 adds the demographic control variables. Migrants send $20 more of the possible lottery prize when their choice will be revealed, which represents an approximate 5 percent increase over the choice not revealed group mean.

Table 3 also reports the coefficients on the demographic control variables included in column 2. Female migrants send on average $26 less than male migrants. Migrants who have been in the United States longer send more, although the effect is small. Migrants who live with a spouse send $29 less than those who do not and migrants in the lowest income bracket are estimated to send $22 less on average than those in the other income brackets. Finally, total annual remittances sent are positively correlated with amount sent in the experiment. The coefficient is small, but it suggests that migrant behavior in the experiment is related to real-world migrant behavior.

The fact that almost all migrants in the not revealed treatment chose to send something and that most chose to send the entire potential prize suggests that the altruistic component of remittances is high. However, the differences between the two treatment groups are evidence that information asymmetries and strategic behavior also play a role. Given that the framework discussed in Section 2 suggests that this strategic behavior will occur when migrants react to costs incurred when sending less than expected, it is instructive to examine whether or not the impact of the information treatment varies in remittance relationships where these costs may be more or less important.

I utilize five variables from the baseline survey as proxies for situations where these costs may be more important: years in the United States, migrant has a child 22 or under in El Salvador, migrant and recipient are closely related, migrant communicates with recipient household weekly, and remittances sent to recipient household. The general motivation for choosing these proxies is that they are indicative of relationships where remittance norms may be higher, migrants may be more likely to have entrusted the recipient with the care of family or investments, and migrants may be more concerned about recipient perceptions of their behavior. A detailed description of the rationale for choosing these variables is provided in Appendix Table 3.^18^ To allow comparability with the binary proxies (child in El Salvador, close relationship, and weekly communication), I create binary measures of the continuous variables (years in the United States and remittances) by splitting the sample at the sample median.^19^ These are certainly not perfect measures, and the analysis is limited by the fact that these characteristics were not randomly assigned and that it was not possible to stratify the information treatment on these characteristics.^20^

To examine how the impact of the monitoring treatment may have varied with each of these proxies, I re-estimate regression Eq. (1), now interacting the treatment indicator with each of the proxy variables. Table 4 presents the results. Each column is a separate regression, reporting the interaction of the treatment with each proxy variable in turn. All regressions also include the main effects of all five proxy variables. The sample size therefore varies slightly from Table 3 because it excludes observations with a missing value for one of these proxies. For each of these variables, the interaction effect is positive. In four of the five columns (all but years in the United States) the interaction effect is larger than the main effect of the treatment, suggesting that treatment effects are concentrated among those migrants where costs are hypothesized to be higher. While these results are suggestive, they must not be over-interpreted: for only two of the five variables are the interaction effects statistically significant.^21^ It should also be noted that while these results are supportive of the notion that migrants send more than they would if they were solely motivated by altruism, I cannot specifically differentiate between the possible strategic motivations, such as the migrant's desire to appear fair or pressure from recipients to send larger amounts home.

The results in Table 3 show that information asymmetries can affect migrants' remittance decisions and that at least some migrants take strategic advantage of a situation where their potential income is not observable to recipients. However, the size of the effect, a 5 percent increase in amount sent, could be considered small. Certainly given the large amounts shared in both treatment groups it seems that other factors (such as altruism) are driving a large part of the remittance decision. However, I argue that the information effect is also economically significant. The size of the effect can be compared to average remittance amounts. Assuming that the average migrant sends remittances on a monthly basis (and the majority of migrants in this sample report doing so), $20 every month results in $240 a year. $240 is approximately equal to the average monthly remittance sent by migrants in this sample, suggesting that the information effect could account for an additional monthly remittance every year. Although the effect size will depend on the amount over which the migrant makes their choice and it is not clear how the magnitudes of the experimental effect would compare to the magnitudes of real life decisions, this comparison does suggest that the information effect can be of economic importance.

Additionally, the effect size is similar to the size of the correlations with the demographic variables in Table 3, meaning that this information effect is of similar importance in determining the migrant's choice as demographic characteristics such as income or gender. The effect size is also comparable to other studies examining the effects of making choices in dictator games known to the recipient in families (Hoel, 2014) and social networks (Leider et al., 2009; Ligon and Schechter, 2012).

### 5.2. Recipient experiment

The migrant experiment found that migrants react strategically to variations in the ability of recipients to monitor their income. Previous literature has suggested that migrant monitoring of recipients should also be important (Ashraf et al., forthcoming; Chen, 2013; de Laat, 2014). To look for these effects in the context of this experiment, I now turn to analysis of the recipient experiment in which recipients allocated a potential $300 remittance prize among four spending categories.

Mean amounts allocated to different spending categories by recipients and migrants are presented in Table 5. The first two columns show the amounts allocated by recipients broken down by the recipient monitoring treatment, and columns 3 and 4 show recipient allocations by the communication treatment. The fifth column shows the means of the preferences reported by the migrant. For both recipients and migrants, education is the most popular choice.^22^ Daily expenses are the next most popular category, followed closely by health and finally restaurant meals. Migrants allocate less to education than recipients and more to the other categories, but no clear evidence indicates that migrants strongly prefer different expenditure categories than recipients.

I utilize the data collected from both the migrant and the recipient to analyze how the treatments affect the pair-level differences between their choices.^23^ Table 6 shows the results from estimating regression Eq. (2). The dependent variables in columns 1 through 4 are the migrant–recipient differences in restaurant spending, education spending, spending on daily expenses, and health spending, respectively. The dependent variable in columns 5 and 6 is the total migrant–recipient difference. Column 6 adds demographic control variables.

The results presented in Table 6 show no evidence of any impact of the recipient monitoring treatment. The coefficients for the four spending categories are too imprecisely estimated to describe a consistent pattern, but for the main outcome of interest, the total migrant recipient difference (columns 5 and 6), the coefficients on both the main effect and the interaction with the communication treatment are close to zero and statistically insignificant.

Differences are evident for the communication treatment. For all spending categories, the coefficient on the main effect of the variable indicating that the migrant preference was revealed to the recipient is negative. This means that the differences between recipient choices and migrant preferences are smaller when the migrants' preferences are revealed than when they are not, even when the recipient choices were not revealed to the migrant. Of all the spending categories, only the difference for education (column 3) is statistically significant, but importantly, so is the total difference (columns 5 and 6), implying that migrant and recipient choices are getting closer together overall. Across specifications the results indicate that there is a $14 reduction in the total difference when the migrant's preferences are revealed and the recipient's allocation will not be shared with the migrant. This represents a 10% reduction relative to the mean in the group where neither the recipient's choice nor the migrant's preference was revealed. The effect is driven by the difference in education spending, largely because education spending is by far the most preferred category.

The first result from the recipient experiment is that the monitoring treatment has no impact on the difference between migrant preferences and recipient choices. This implies that recipients do not react strategically to variation in whether or not their choices will be observed. The framework presented in Section 2 proposes an explanation for why recipients may not take advantage of the opportunity to hide their spending choices from migrants. The costs that would induce recipients to react to being monitored may simply be low across the population. In practice, this would result in a situation where recipients are not compelled to spend remittances according to migrant preferences beyond what they choose to do altruistically.

Although low costs associated with remittance spending decisions are a convincing explanation for the lack of effect of the monitoring treatment, it is important to consider other possible explanations. The first alternative explanation is that migrant monitoring of recipients is essentially perfect and that recipients know that their choices will be discovered if they win. However, only 24% of migrants correctly report student GPA and 43% correctly report how students travel to school (Table 1); therefore it does not seem plausible that existing monitoring is good enough to render the experimental variation irrelevant. A second explanation is that migrants and recipients have the same preferences for spending, and therefore they make the same choices regardless of punishment ability. This may be true for some families, but if it were true for most, there should be no impact of the communication treatment. Additionally, only 48% of migrant–recipient pairs report the same three budget priorities (Table 1), further evidence of heterogeneity in preferences. A final alternative explanation is that migrants simply have no preference for how the remittances are spent and allow recipients to spend them however they see fit. This cannot be definitively ruled out, but again, if recipients knew that migrants had no strong preferences, there should be no impact of the communication treatment.

The second result from the recipient experiment is that revealing migrant preferences decreases the difference between migrant preferences and recipient choices even when the recipient choice will be not be revealed to the migrant. This suggests that migrant preferences do matter to recipients and that some deviation from those preferences may be inadvertent. A potential criticism of this result is that recipients are simply reacting to being given a suggested allocation for the choice they are making and may have reacted in the same way even if the preferences were attributed to someone besides the migrant. I address this concern by examining heterogeneity in the effects of the communication treatment by proxies for the quality of information in the relationship. Specifically, in Appendix Table 5, I estimate regression Eq. (2) separately by whether or not the migrant can correctly report the student's GPA and mode of transport to school. Although these variables are not direct representations of recipient knowledge of migrant preferences, they are likely to indicate low information quality in general. If recipients are reacting to a lack of knowledge of the migrants' preferences, the effects of the communication treatment should be concentrated among pairs where information quality is low. I find suggestive evidence that this is indeed the case: effects of the communication treatment appear to be concentrated among migrants who do not know students' GPAs or modes of transportation to school.^24^

### 5.3. Threats to interpretation

Although the experimental methodology used in this paper allows for the causal identification of the effects of information asymmetries, it is important to consider how the experimental context could distort participant behavior relative to real life. The first potential issue is experimenter-demand effects, the idea that because participant decisions in the experiment are measured by the experimenter participants may act in a more pro-social manner than they would if their choices were truly anonymous. This question has been widely explored through a series of lab experiments that vary whether or not participant choices are observable to the experimenter. Levitt and List (2007) provide a review of these studies which generally show that subjects become less generous as anonymity is increased, but Barmettler et al. (2012) find no impact of experimenter–subject anonymity when using a new technique that resolves their criticisms of past studies. However, experimenter-demand effects are actually broader than the issue of experimenter–subject anonymity, as subjects may behave differently simply because they know they are participating in an experiment (Levitt and List, 2007). This issue is certainly present in this study, but because the questions were framed as a reward for participating in the survey and not explicitly as an experiment, it is possible that the scrutiny is not as important here as in a true lab experiment. Regardless, because the focus of the paper is the effect of the information treatment, the main results are only affected if experimenter-demand effects are expected to be more important for one treatment group than for the other. While it is not possible to rule this out, there is no clear argument for the direction of such an interaction effect.

Experimenter-demand effects can also play a role in the recipient communication treatment. Recipients may, for example, feel obliged to follow migrant preferences because the surveyor will observe their choice. Again, there is no way to rule this out, but given that there is no impact of the migrant monitoring treatment, in order for experimenter-demand effects to explain a large portion of the observed effect of the communication treatment, recipients would have to respond much more strongly to scrutiny by the surveyors than to observation by the migrants themselves.

The second issue is that the prize money in the experiment is a onetime windfall as opposed to regular earned income. Studies that have examined earned versus unearned windfall income have found that people are more generous with unearned winnings (for example Cherry et al. (2002) and Jakiela (2014)). As in the case of experimenter-demand effects, the ultimate concern in this experiment is whether or not this windfall effect differentially impacts the two treatment groups. In this case, the impact of the monitoring treatment may increase if income were earned as migrants feel more ownership over the winnings but are still compelled to share them when their choices are observable. Alternatively, if social norms dictate that migrants share more of windfall income than earned income, a larger effect of monitoring would be expected with windfall income. Both of these effects may also be operating at the same time and given that there is no way to identify which might larger, the results should be interpreted with this in mind.

The final issue is that of the fungibility of choices made during the experiment. Both migrants and recipients could potentially undo their choices during the experiment through their actions afterward. For example, migrants could choose to not send a remittance that they would have sent otherwise. Although some of this behavior may be occurring, it is not necessarily important for the interpretation of the treatment. If the results show differences between the two treatments then that is evidence that people are reacting to variations in information.^25^

## 6. Conclusion and policy implications

This paper analyzes a set of experiments designed to test for the effects of information asymmetries in transnational households. Economic studies of information asymmetries in households with migrants have until now focused on migrant monitoring of recipient behavior and the impacts of offering migrants greater control over how remittances are spent. This is the first study that explicitly looks at the effect of information asymmetries on *both* sides of the remittance relationship—migrants' sending of remittances as well as recipients' spending of those remittances. Despite the previous emphasis on migrant monitoring, the results of the two monitoring treatments presented in this paper are that, in this context, only migrants strategically react to variations in the probability that their actions will be monitored.

This is an important finding, not only because it shows that information asymmetries have an important impact on the remittance sending decision, but also because this suggests that recipients have important influence in the migrant–recipient relationship.^26^ Because migrants control the flow of remittances they send to their family members, documenting this recipient influence is important precisely because it goes against the prior that those who control the money are necessarily in the best bargaining position. Policymakers who seek to design tools to facilitate the sending of remittances and enhance their impacts should consider the role of the recipient in determining remittance amounts.

The analysis in this paper indicates that because migrants are responding to the opportunity to hide income, some of them are already sending home more than they would choose to altruistically. This implies that programs that seek to further increase remittances may face difficulties within this group. Policymakers should also consider the welfare implications of such a policy. The pressure that migrants face to send remittances is related to a growing literature on sharing pressures in family networks in developing countries. Recent work by di Falco and Bulte (2011) and Jakiela and Ozier (2012) suggests that the expectation that resources will be shared with extended family may inhibit individual economic progress. In this context, given the results of the migrant experiment and the low income status of the migrants, it is not clear that potential extra remittance funds are necessarily more welfare enhancing from the perspective of the migrant when spent in El Salvador than if they were to be used by the migrants in the United States.

Overall, the findings that information asymmetries can affect both the sending and spending of remittances suggest that interventions or technological innovations that improve communication in transnational households could have important effects on financial decisions made by both migrants and recipients. In particular, the results of the communication experiment imply that for migrants who wish to change the spending behavior of their family members, policies that improve communication about spending preferences may be an inexpensive way to achieve a higher level of compliance with their preferences.

This discussion assumes that alleviating inadvertent information asymmetries in transnational households increases welfare. However, if, for example, migrant preferences are based on old or misinformation, encouraging the recipient to follow such preferences may actually have negative impacts on the recipient household. This, combined with the strategic behavior of migrants in the migrant experiment, suggests that the welfare effects of alleviating information asymmetries are complex. While this paper documents the importance of these information issues, further research should more carefully investigate their welfare implications in both transnational and coresident households.

## Supplementary Material

Supplement

## Figures and Tables

**Table 1:** Baseline summary statistics.

	All observations			Observations with completed recipient survey		
		
	Mean	SD	N	Mean	SD	N
*Baseline variables from migrant survey*						
Migrant is female	0.50	0.50	1581	0.51	0.50	1298
Migrant age	36.83	9.41	1538	36.92	9.29	1264
Migrant can read and write	0.96	0.20	1554	0.96	0.20	1275
Migrant's years of education	9.08	4.67	1560	9.01	4.67	1282
Migrant's years in the US	11.31	6.38	1577	11.13	6.27	1295
Migrant is married	0.62	0.48	1575	0.63	0.48	1294
Migrant lives with spouse	0.49	0.50	1579	0.50	0.50	1296
Migrant's total number of children	2.28	1.69	1579	2.34	1.69	1296
Migrant's number of children in El Salvador	1.01	1.43	1577	1.07	1.47	1294
Migrant's number of children in US	1.26	1.32	1575	1.25	1.29	1293
Migrant's hh size in US	4.32	1.98	1581	4.36	1.96	1298
Migrant has child 22 or under in El Salvador	0.32	0.47	1581	0.34	0.47	1298
Recipient is migrant's close relative	0.29	0.45	1574	0.31	0.46	1291
Migrant has worked in last 12 months	0.89	0.31	1581	0.89	0.31	1298
Migrant in lowest income bracket	0.52	0.50	1429	0.53	0.50	1181
Migrant sent remittances to recipient hh	0.85	0.36	1580	0.87	0.34	1297
Migrant's annual regular remittances to recipient hh ($)	2298	2907	1565	2440	2998	1283
Migrant's annual irregular remittances to recipient hh ($)	337	706	1575	344	707	1293
Migrant's annual total remittances to recipient hh ($)	2629	3199	1563	2777	3284	1281
Migrant's annual total remittances to other hhs ($)	1097	1905	1567	1123	1944	1284
Migrant communicates with recipient hh at least weekly	0.69	0.46	1578	0.71	0.45	1295
*Baseline variables from recipient survey*						
Recipient is target student				0.45	0.50	1298
Recipient is student's guardian				0.40	0.49	1298
Recipient is female				0.68	0.47	1298
Recipient age				34.20	15.84	1295
Recipient is married				0.36	0.48	1298
Recipient's years of education				9.37	5.27	1292
Recipient lives in urban area				0.43	0.50	1298
Recipient's hh size				4.99	2.04	1296
Annual remittances received from migrant ($)				1522	1916	1203
*Baseline comparison variables*						
Migrant and recipient report same hh budget priorities				0.48	0.50	1231
Migrant and recipient report same student GPA				0.24	0.43	1041
Migrant and recipient report same student mode of transport				0.43	0.50	1107

Notes: Samples are observations with non-missing data for questions in the migrant experiment. Completed recipient survey sample additionally conditions on completion of the recipient survey and non-missing migrant and recipient information for questions in the recipient experiment. The number of observations varies slightly with missing values. Recipient is defined as a close relative if migrant reports recipient to be spouse, parent or child. Migrants in the lowest income bracket chose $400 or less as the weekly income of themselves plus their coresident spouses. The other categories were $401–600, $601–800, and $801 and above. Annual regular remittances were collected by asking for the frequency of remittances sent and the average amount sent each time. Annual irregular remittances are remittances sent for special occasions or emergencies. The recipient variables in all cases refer to the person completing the recipient survey. The baseline comparison variables were asked on both surveys and are equal to one if the migrant and recipient responses match. Both respondents were asked to choose the three most important budget priorities for the recipient household from a list of seven categories. Student refers to the student identified by the migrant during the baseline survey. GPA and mode of transport were only asked when the student was reported to be in school.

**Table 2:** Balance tests.

	Migrant experiment			Recipient experiment: monitoring treatment			Recipient experiment: communication treatment		
			
	Treatment group means: migrant choice…		P-value for difference of means	Treatment group means: recipient choice…		P-value for difference of means	Treatment group means: migrant choice…		P-value for difference of means
			
	…Not revealed to recipient	…Revealed to recipient		…Not revealed to migrant	…Revealed to migrant		…Not revealed to recipient	…Revealed to recipient	
*Attrition*									
Recipient survey completed	0.82	0.83	0.819	0.81	0.83	0.315	0.82	0.83	0.730
*Baseline variables from US survey*									
Migrant is female	0.53	0.49	0.165	0.52	0.50	0.532	0.49	0.53	0.186
Migrant age	36.90	36.94	0.941	36.56	37.27	0.176	36.90	36.95	0.922
Migrant can read and write	0.95	0.97	0.150	0.95	0.96	0.461	0.96	0.95	0.295
Migrant's years of education	9.01	9.00	0.966	9.02	9.00	0.947	8.97	9.04	0.798
Migrant's years in the US	10.90	11.37	0.178	11.18	11.08	0.774	11.13	11.13	0.993
Migrant is married	0.61	0.65	0.151	0.65	0.61	0.175	0.63	0.63	0.952
Migrant lives with spouse	0.50	0.50	0.956	0.51	0.49	0.543	0.50	0.50	0.957
Migrant's total number of children	2.34	2.34	0.956	2.30	2.38	0.352	2.37	2.31	0.560
Migrant's number of children in El Salvador	1.03	1.10	0.365	1.01	1.12	0.206	1.04	1.09	0.557
Migrant's number of children in US	1.28	1.22	0.410	1.27	1.24	0.725	1.31	1.20	0.105
Migrant's hh size in US	4.34	4.38	0.720	4.43	4.29	0.183	4.43	4.29	0.214
Migrant has child 22 or under in El Salvador	0.32	0.37	0.059	0.33	0.35	0.366	0.34	0.34	0.885
Recipient is migrant's close relative	0.29	0.33	0.178	0.30	0.32	0.539	0.34	0.29	0.059
Migrant has worked in last 12 months	0.90	0.89	0.943	0.89	0.90	0.401	0.89	0.89	0.950
Migrant in lowest income bracket	0.53	0.53	0.886	0.51	0.54	0.229	0.53	0.53	0.934
Migrant sent remittances to recipient hh	0.87	0.86	0.586	0.86	0.88	0.510	0.87	0.87	0.802
Migrant's annual regular remittances to recipient hh ($)	2494	2386	0.520	2435	2444	0.953	2315	2561	0.141
Migrant's annual irregular remittances to recipient hh ($)	354	334	0.627	382	308	0.062	353	335	0.655
Migrant's annual total remittances to recipient hh ($)	2828	2726	0.579	2802	2752	0.786	2648	2903	0.165
Migrant's annual total remittances to other hhs ($)	1059	1185	0.245	1137	1110	0.804	1068	1177	0.314
Migrant communicates with recipient hh at least weekly	0.73	0.69	0.057	0.73	0.69	0.192	0.70	0.72	0.585
*Baseline variables from recipient survey*									
Recipient is target student	0.45	0.45	0.907	0.44	0.46	0.402	0.46	0.44	0.495
Recipient is student's guardian	0.42	0.38	0.160	0.42	0.38	0.239	0.39	0.41	0.319
Recipient is female	0.69	0.67	0.331	0.68	0.68	0.998	0.68	0.68	0.726
Recipient age	35.09	33.31	0.043	34.44	33.97	0.589	34.29	34.11	0.835
Recipient is married	0.36	0.36	0.941	0.41	0.32	0.001	0.35	0.38	0.243
Recipient's years of education	9.21	9.54	0.285	9.22	9.53	0.294	9.30	9.45	0.622
Recipient lives in urban area	0.43	0.44	0.649	0.41	0.46	0.061	0.42	0.45	0.312
Recipient's hh size	4.90	5.08	0.111	5.04	4.95	0.471	5.06	4.93	0.271
Annual remittances received from migrant ($)	1491	1553	0.580	1534	1510	0.825	1484	1559	0.497

Notes: Samples are observations with non-missing values for the experiment questions and completed recipient survey. Attrition is measured from sample of all migrants who completed the survey and the migrant experiment to sample with completed recipient survey and recipient experiment. Sample size for each comparison of means varies slightly by missing values for each variable. The percentage of missing values for each variable is also tested for balance across treatment groups with no significant differences. Other notes on variable construction are as in Table 1. P-values come from a regression of each variable on treatment, with standard errors adjusted for heteroskedasticity.

**Table 3:** Impact of monitoring treatment on migrant remittance decision.

	(1)	(2)
	
	Dependent variable: amount sent by migrant	
Migrant choice revealed to recipient	20.40** [10.27]	19.50* [10.26]
Migrant is female		−26.46** [11.09]
Migrant age		−0.487 [0.741]
Migrant's years of education		−0.119 [1.225]
Migrant's years in the US		1.968* [1.071]
Migrant lives with spouse		−28.75** [11.83]
Migrant's hh size in US		1.293 [2.800]
Migrant has child 22 or under in ES		0.984 [12.41]
Recipient is migrant's close relative		−0.675 [12.74]
Migrant in lowest income bracket		−21.73* [12.65]
Migrant's annual total remittances to recipient hh		0.00319* [0.00192]
Migrant communicates with recipient hh at least weekly		−1.122 [12.68]
Observations	1298	1298
R-squared	0.133	0.159
Mean in migrant choice not revealed to recipient	441.4	

Notes: Robust standard errors in brackets. Samples are observations with non-missing values for all experiment questions and completed recipient survey. Amount sent by migrant is the amount that migrants chose to send when splitting $600 between themselves and recipients. All regressions include stratification group fixed effects: dummy variables for the groups of survey numbers within which randomization was stratified. Recipient is defined as close relative if migrant reports recipient to be his spouse, parent or child. Migrants in the lowest income bracket chose $400 or less as the weekly income of themselves plus their co-resident spouses. The other categories were $401–600, $601–800 and $801 and above. Annual total remittances are the combination of regular and irregular remittances. Annual regular remittances were collected by asking for the frequency of remittances sent and the average amount sent each time. Annual irregular remittances are remittances sent for special occasions or emergencies.

*** p < 0.01,

** p < 0.05,

* p < 0.1.

**Table 4:** Impact of monitoring treatment on migrant remittance decision: interactions.

	(1)	(2)	(3)	(4)	(5)
	
	Dependent variable: amount sent by migrant				
	
	Proxy variable is…				
	
	Years in the United States: below sample median	Migrant has child 22 or under in El Salvador	Recipient is close relative of migrant	Migrant communicates with recipient household weekly	Migrant's annual remittances to recipient household: above sample median
Migrant choice revealed to recipient	18.16 [17.24]	9.769 [13.26]	12.91 [13.29]	9.994 [20.98]	−1.112 [16.38]
Migrant choice revealed * proxy	6.888 [22.60]	49.52** [24.45]	21.62 [23.53]	14.44 [24.69]	36.41* [22.22]
Observations	1268	1268	1268	1268	1268
R-squared	0.225	0.219	0.214	0.212	0.214
Mean in migrant choice not revealed & proxy = 0	462.4	449.3	445.6	451.7	447.2
Mean in migrant choice not revealed & proxy = 1	427.0	424.3	433.9	438.4	437.4
Main effects for all five proxy variables	Yes	Yes	Yes	Yes	Yes

Notes: Robust standard errors are in brackets. Samples are observations with non-missing values for all experiment questions, completed recipient survey and non-missing values for the proxy variables. Amount sent by migrant is the amount that migrants chose to send when splitting $600 between themselves and recipients. All regressions include stratification group fixed effects: dummy variables for the groups of survey numbers within which randomization was stratified. The proxy variable is interacted with all other variables in the regression (treatment, other proxies, and stratification cells). Recipient is defined as close relative if migrant reports recipient to be his spouse, parent or child. Annual total remittances are the combination of regular and irregular remittances. Annual regular remittances were collected by asking for the frequency of remittances sent and the average amount sent each time. Annual irregular remittances are remittances sent for special occasions or emergencies. The median years in the US is 10 and the median remittances sent to the recipient household are $1800.

*** p < 0.01,

** p < 0.05,

* p < 0.1.

**Table 5:** Mean amounts allocated to spending groups by recipients and migrants: recipient experiment.

	Means of recipient choices by treatment group:				Means of migrant preferences:
	
	Monitoring treatment		Communication treatment		
		
	Recipient choice not revealed to migrant	Recipient choice revealed to migrant	Migrant preferences not revealed to recipient	Migrant preferences revealed to recipient	
*Amount allocated to*:					
Restaurant meals	6.11	5.46	5.38	6.17	11.74
Education	175.54	166.22	170.97	170.64	141.41
Daily expenses	66.05	75.59	72.85	68.99	76.56
Health expenses	52.30	52.73	50.80	54.20	70.28
*Observations*	638	660	641	657	1298

Notes: Samples are observations with non-missing values for all experiment questions and completed recipient survey. Means in columns 1 through 4 are from responses by recipients when asked to allocate $300 across four spending categories. Means in column 5 are responses from migrants when asked how they would like the recipient to allocate the funds.

**Table 6:** Impact of monitoring and communication treatments on recipient allocation decision.

	(1)	(2)	(3)	(4)	(5)	(6)
	
	Dependent variable: migrant–recipient difference in…				Dependent variable: total migrant–recipient difference	
	
	Restaurant spending	Education spending	Daily expenses spending	Health spending		
Recipient choice revealed to migrant	−2.848 [3.276]	−0.0745 [7.036]	9.540 [5.888]	−2.380 [5.996]	2.119 [6.619]	3.158 [6.714]
Migrant preference revealed to recipient	−4.591 [3.054]	−18.55*** [6.886]	−0.610 [6.061]	−5.267 [5.994]	−14.51** [6.752]	−13.86** [6.918]
Choice revealed * preference revealed	3.633 [4.202]	8.570 [9.951]	−10.88 [8.482]	2.218 [8.415]	1.769 [9.600]	0.340 [9.745]
Observations	1298	1298	1298	1298	1298	1298
R-squared	0.102	0.105	0.093	0.091	0.105	0.122
Mean in recipient choice not revealed, migrant preference not revealed	18.0	116.2	78.3	77.9	145.2	
Control variables	No	No	No	No	No	Yes

Notes: Robust standard errors in brackets. Samples are observations with non-missing values for all experiment questions and completed recipient survey. Dependent variables are the absolute difference between the recipient's choice and the migrant's preferences in each category. The total difference is the sum across the four difference variables for each observation, divided by two. All regressions include stratification group fixed effects: dummy variables for the groups of survey numbers within which randomization was stratified and treatment status in the migrant experiment. Control variables are migrant and recipient gender, age, years of education, and household size. Controls also include migrant years in the United States, whether migrant lives with spouse, whether migrant has a child 22 or under in El Salvador, whether the migrant and recipient are close relatives, if the migrant is in the lowest income bracket, annual total remittances to recipient household, whether the migrant and recipient communicate at least weekly, and the number of days in between migrant and recipient survey.

*** p < 0.01,

** p < 0.05,

* p < 0.1.

## Footnotes

1 Exceptions include Bloch and Rao (2002) and Chen (2013).

2 Recruitment methods and locations were very similar to those successfully used in Ashraf et al. (forthcoming).

3 The dynamics of transfer arrangements in extended family networks has been studied (Foster and Rosenzweig, 2001), but little is known about how information imperfections affect behavior in these arrangements. The notable exception is Jakiela and Ozier (2012), but the main focus of that paper is investment decisions, as opposed to sharing and spending decisions.

4 Torero and Viceisza (2013) and De Arcangelis et al. (2014) also play remittance dictator games, but the focus of those studies was migrant control of remittance spending.

5 This idea of altruistic remittances coexisting with other motives is well explored in the literature. See for example, Lucas and Stark (1985). Rapoport and Docquier (2006) review the economic literature on remittance motivations and Carling (2014) synthesizes the ethnographic literature.

6 For ease of exposition, I will use the term cost throughout the text, but the reader should keep in mind that this cost could be negative (i.e. a benefit).

7 This observation of income will likely occur through the migrant's social and family network. Given that the networks within which migrants in the United States live and work are often closely related to their home country networks (Munshi, 2003), instances when the probability that income will be observed is quite high are likely.

8 This was followed by the offer of a product designed to facilitate the sending of remittances for education. This was a randomized intervention, and migrants received offers of different versions of the product depending on their assigned treatment group. Migrants in a control group received only information and no product offer.

9 Surveyors report very few cases of participants requesting this information. The first prize was awarded midway through survey work, and the second when survey work had concluded. Migrants were eligible for only one drawing.

10 As noted earlier, two migrant prizes were given out. Participants were informed of the number of prizes and the dates of the drawings if they asked. Surveyors report that very few participants inquired, so in practice most participants did not know the odds of winning.

11 This method should not be confused with the common method used in economic experiments in which participants complete a number of different tasks and one is randomly chosen to be paid out.

12 Despite its widespread use, it is important to consider how the lottery mechanism may affect respondent choices. Studies that directly examine how using lotteries affects choices have yielded mixed results. Baltussen et al. (2012) find that subjects appear to be less risk averse when lotteries are used and Sefton (1992) finds that subjects are more generous. Conversely, Bolle (1990) finds no difference in decisions between guaranteed payouts and those determined by lottery. However, all of this work has examined choices under low to medium stakes; there has been no systematic study comparing guaranteed payoffs to lottery payoffs at high stakes similar to the ones used in this paper. Laury (2005) shows that respondents make the same choices when low stakes payoffs are random as when payoffs are guaranteed. However, a separate treatment in which payoffs were random but stakes were increased by ten did change behavior, suggesting that calibrating the face value of the prize may be the most important consideration.

13 The time at which this interaction occurred relative to the other components of the experiments is depicted in Figs. 1 and 2.

14 Surveyors report very few cases of participants requesting this information. Two prizes were awarded midway through survey work and the other two when survey work had concluded. Recipients were eligible for only one drawing.

15 14% refused, 7% were not from El Salvador, and 2% had other reasons.

16 The success of this strategy depends on migrant preferences being balanced at baseline. There are no significant differences between treatment groups for either recipient treatment for migrant responses across the four spending groups.

17 The p-value on the two-sample Wilcox rank-sum for equality of distributions is 0.034.

18 Given that these variables generally indicate a stronger or closer relationship between migrant and recipient, arguably they may also be proxies for higher levels of pure altruism. However, the nature of altruistic remittances is such that they should not be affected by variations in monitoring. In other words, if these variables were proxies for only altruism, the treatment effect of monitoring in the high-altruism subgroups should not be higher than in low-altruism subgroups.

19 To be consistent with the direction of the hypothesized correlations of the other proxies, below median years in the United States has been coded as one and above median years in the United States has been coded as zero.

20 Despite the inability to stratify randomization on these variables, Appendix Table 4 shows that the subsamples are largely balanced on baseline characteristics.

21 An alternative method of evaluating the differential effect of the treatment in these different groups is to include them all in one regression and interact each one with the treatment indicator. This has the benefit of showing whether each of these effects exists while controlling for the others. Unfortunately, in this specification, I do not have the power to say anything definitive about any of the interaction effects. Three of the five (years in the United States, migrant has a son or daughter 22 or under in El Salvador, and annual remittances) are large and therefore consistent with the patterns in Table 4. Some collinearity is present however, as the interactions of treatment with close relative and weekly communication are close to zero.

22 It should be noted that the preference for education may be partly due to the fact that participants answered this question at the conclusion of a survey that focused on questions about education, meaning that they may have been primed to consider education. This is not necessarily a problem as there is no reason to believe that either migrants or recipients were more primed than the other, but it does suggest that the indicated preferences should be considered as only in the context of the experiment.

23 Appendix C discusses the results of an alternative specification that examine the impacts of the monitoring and communication treatments on the average amount allocated to each category.

24 A potential caveat to this argument is that the migrant lack of knowledge may in fact represent lower baseline valuation of education on behalf of the migrant, which could be correlated with larger initial differences in preferences. However, migrants in the low information groups do not allocate less money to education.

25 Even without evidence of differences between treatments, if participants wish to do something that their partner would disagree with, it makes sense for them to take advantage of the experiment to do so, when the probability of keeping that action secret is high.

26 As described earlier this influence may operate directly, through pressure from the recipient, or indirectly, if the recipient wishes to appear altruistic to the recipient.

## Appendix A. Details of companion experiment

The experiments described in this paper were conducted as a part of the data collection for a field experiment on remittances and education described in detail in “Channeling Remittances to Education: A Field Experiment among Migrants from El Salvador” (Ambler et al., forthcoming). This appendix provides details on the implementation of that project and discusses the potential ways in which the design may have impacted the results in this paper.

The goal of the companion project was to study a new payment mechanism called the EduRemesa that allowed migrants to direct remittance funds to the education of a secondary or tertiary student in El Salvador. The project studied both the unsubsidized and subsidized versions of the EduRemesa, analyzing both take-up of the product and its impacts on education related outcomes of beneficiary students.

The EduRemesa was administered by FEPADE, an educational foundation in El Salvador and provided funds directly to the beneficiary student for each month of a ten month school year. These funds were allocated through a bank account and debit card provided to the student for this purpose. Migrants chose from predetermined EduRemesa amounts: $300 or $500 for secondary students and $600 or $800 for tertiary students. These funds were deposited in equal installments across the ten month school year. EduRemesas were sent by the migrant through Viamericas, a money transfer company. The money was remitted to FEPADE, and the migrants indicated the student as the beneficiary in the course of the transaction.

Migrants were recruited to participate by project staff stationed in the two Washington, DC area locations of the Salvadoran consulates. Because the EduRemesa was aimed at secondary and tertiary level students, migrants were required to name a student at that level in order to participate. In particular, because the study sought to measure project impacts on students in El Salvador through surveys, we elicited the name of a student who the migrant would be likely to support if offered the EduRemesa from all migrants in all treatment groups. This elicitation was done at the beginning of the survey before any treatments were administered so that the choice of student would not be affected by the treatment offer. Migrants were informed that they could enter one student in El Salvador of their choosing into a lottery to win a $500 scholarship for the next academic year. The named student and their household are referred to as the target student and the target household respectively.

Migrants who agreed to participate completed a baseline survey that contained questions related to demographics, remittances, and the target student and household. At the end of the survey the migrant answered the two experimental questions analyzed in this paper, first making the allocation of the potential $600 lottery prize and then listing their preferences for the recipient spending of the potential $300 recipient lottery prize. Following the conclusion of the survey, the project staff implemented the randomized EduRemesa treatment. Because the implementation of the treatment occurred after the migrant answered the experimental questions, it should not have impacted the responses to those questions.

The EduRemesa treatment was allocated through a two-stage randomization process. First, each day and each recruitment location was randomized into either the control group or the group which received an offer of the EduRemesa. Within the group that received the EduRemesa offer, an individual-level randomization was performed to allocate each migrant into one of three subsidy groups: those receiving no matching funds, those for whom EduRemesa funds provided by the migrant were matched one to one by the project, and those for whom those funds were matched three to one.

Migrants in the control group received information discussing the importance of sending remittances home for education which highlighted the strategy of sending money directly to the student in small, monthly installments. Those in the EduRemesa treatments received this information, as well as the introduction to the EduRemesa product. At the conclusion of the presentation of the product, migrants in the EduRemesa treatment groups were asked if they were interested in participating and whether they would like to receive follow-up information. Migrants who expressed an interest were re-contacted by the survey team to assist with the process and answer any further questions. The survey team continued to follow up until the migrant indicated that he or she was no longer interested.

During the baseline survey the migrant was required to provide contact details for the target student or household. Within a week after the initial interaction, this information was used to contact the target household and perform an initial survey. These interviews were conducted with the target student if the target student was 18 or over, and the target student's guardian if the target student was under 18. If neither was available, the interviews were conducted with another knowledgeable adult in the household. This survey contained questions related to demographics and remittances, and had an extensive module related to education expenditures for all students in the household. The experimental question analyzed in this paper (the recipient decides how to allocate a potential $300 lottery prize) was the last question in the survey and ended the interaction. No information related to the EduRemesa was discussed during the interview and surveyors did not know which version of the EduRemesa the migrant in the family had been offered if at all.

The most important way in which the design of the EduRemesa study could have impacted the results in this paper relates to the time between when the baseline survey with the migrant was conducted and when the target household was interviewed. The median number of days between interviews was eight, and given that the randomized EduRemesa treatment was implemented at baseline in the United States, there was time for migrants to discuss it with their family members before completing the interview.

This could have affected the recipients' responses by priming them to think about education and therefore allocate more money to educational expenditures. This is certainly possible, but there is no way to directly test for this. In fact, the survey itself focused heavily on education, so it is likely that played a larger part in priming respondents. In order for this to threaten the interpretation of the results in the paper (which focus on differences between the migrant and the respondent, not the particular category to which they allocated money), the EduRemesa treatments must have had a *differential* impact on respondent choices. However, including the EduRemesa treatment variables in the regressions has no impact on the results, and they are additionally not statistically significantly correlated with the outcome variables. Finally, the EduRemesa treatments are not significantly related to the raw allocation choices made by the recipients in any systematic way. For example, recipients do not allocate more money to education in cases where the migrant received the three to one subsidy offer.

## Appendix B. Text used in experiments

### B.1. Migrant experiment

To thank you and your family for your participation in this study now we are going to give you the opportunity to participate in two more lotteries. Let me tell you about them.

Question 1:

First, you have the chance to win $600. You can keep this money or you can choose to send some or all of it to *name of person to be surveyed* in El Salvador. However, you must tell me now how much you want to keep and how much you want to send and if you win the choice you make now will be carried out.

**Treatment 0**: Keep in mind that because of the rules of this project we cannot inform *name of person to be surveyed* about what you decide to dowith the money. This means that your decision is a secret. *Name of person to be surveyed* will not be told how much you have decided to send and how much you have decided to keep.

**Treatment 1**: Keep in mind that because of the rules of this project we have to inform *name of person to be surveyed* about what you decide to do with the money. This means that your decision is not a secret. *Name of person to be surveyed* will be told how much you have decided to send and how much you have decided to keep.

Let's make this decision now. You have the following options: (*surveyor shows options to migrant*)

- KEEP: $600 and SEND: $0
- KEEP: $500 and SEND: $100
- KEEP: $400 and SEND: $200
- KEEP: $300 and SEND: $300
- KEEP: $200 and SEND: $400
- KEEP: $100 and SEND: $500
- KEEP: $0 and SEND: $600

Question 2:

Now I am going to tell you about a second lottery that is completely different and separate from the first one. Because you have participated in our survey, *name of person to be surveyed* will have the opportunity to win a remittance worth $300 and will need to choose how he/she would like to receive it if he/she wins. He/she cannot pick anything but must choose among the following categories: meals at local restaurants, education related expenses, daily expenses like groceries, and health related expenses. He/she can spend it all on one thing or break it up among different things.

*Name of person to be surveyed* will decide how he/she would like to receive the remittance. However, we would like to know how you would prefer that *name of person to be surveyed* allocate this remittance.

**Table:**

Spending category:	Amount:
1. Meals at local restaurants (ex: Pollo Campero, Burger King)	
2. Education related expenses (ex: supplies, uniforms, books)	
3. Daily expenses like groceries	
4. Health related expenses (ex: medicine, doctor's visits)	
Total (verify adds up to $300):	

### B.2. Recipient experiment

Because *name of migrant* participated in our study, you now have the chance to receive a remittance worth $300. Some participants like you will be chosen to receive this remittance. However, this remittance can only be spent on a limited number of things. In order to participate you must tell me now how you would like to allocate the remittance among the following categories, and if you win, you will receive exactly what you have told me that you want. The categories are: meals at local restaurants, education related expenses, daily expenses like groceries, and health related expenses. You can spend it all on one thing or break it up among different things.

**Treatment 1**: You can choose anything that you like.

Keep in mind that because of the rules of this project we cannot inform *name of migrant* about what you decide to do. This means that your decision is a secret. *Name of migrant* will not be told about what you decide to spend the money on.

**Treatment 2**: When we spoke with *name of migrant* we asked him/her what he/she prefers for you to spend this money on and he/she indicated that he/she would like you to choose ______. However, you can choose anything that you like.

Keep in mind that because of the rules of this project we cannot inform *name of migrant* about what you decide to do. This means that your decision is a secret. *Name of migrant* will not be told about what you decide to spend the money on.

**Treatment 3**: You can choose anything that you like.

Keep in mind that because of the rules of this project we have to inform *name of migrant* about what you decide to do. This means that your decision is not a secret. *Name of migrant* will be told about exactly what you decided to spend the money on.

**Treatment 4**: When we spoke with *name of migrant* we asked him/her what he/she prefers for you to spend this money on and he/she indicated that he/she would like you to choose ______. However, you can choose anything that you like.

Keep in mind that because of the rules of this project we have to inform *name of migrant* about what you decide to do. This means that your decision is not a secret. *Name of migrant* will be told about exactly what you decided to spend the money on.

Let's make this decision now. How would you like to allocate this remittance among the following categories?

**Table:**

Spending category:	Amount:
1. Meals at local restaurants (ex: Pollo Campero, Burger King)	
2. Education related expenses (ex: supplies, uniforms, books)	
3. Daily expenses like groceries	
4. Health related expenses (ex: medicine, doctor's visits)	
Total (verify adds up to $300):	

## Appendix C. Alternative specification for recipient experiment

This appendix discusses an alternative specification to analyze the impacts of the recipient treatment that does not utilize information on the migrants' preferences and examines the impacts of the treatments on the amount allocated by the recipient to each category. The results are presented in Appendix Table 6. This specification finds no impact of the communication treatment. As discussed in the estimation section of the main text, this is possible if average migrant preferences are similar to average recipient preferences but pair-level disagreement in those preferences is important.

An interesting component of this specification is that the monitoring treatment does appear to increase the average amount allocated to daily expenses. However, the interaction term between the two treatments is negative, suggesting that recipients may have thought migrants wanted them to spend more on daily expenses, but realized they were incorrect when informed of migrant preferences. There is also a decrease in the amount allocated to education, although this effect is not statistically significant. This pattern of results would be compatible with the results in Table 6 of the main text if recipients respond to the monitoring treatment by altering their choices, and as a result some migrant–recipient differences decrease and others increase. One would expect this to happen only when some recipients are not informed about migrant preferences (causing them to possibly make a choice that increases the difference in preferences). Given that the communication treatment makes migrant preferences explicit, if migrants are indeed responding to the monitoring treatment we should see a decrease in the migrant–recipient difference for those recipients who are exposed to both the monitoring and the communication treatment. However, there is no interaction effect of the two treatments in the specification presented in Table 6. Given this, the interpretation of these results of the monitoring treatment is unclear. As the recipient experiment was specifically designed to measure changes in the pair-level differences, I focus on those results.

## Appendix D. Appendix Tables

**Appendix Table 1:** Comparison of migrants in study with DC-area Salvadorans in the American Community Survey.

	Baseline survey	American Community Survey: 2008–201 0 3-year sample
		
	Analysis sample	Salvadoran-born, not US citizen
Migrant is female	0.51	0.46
Age of migrant	36.92 [9.30]	36.05 [10.39]
Migrant's years in the US	11.13 [8.09]	12.93 [7.89]
Migrant's hh size in the US	4.36 [1.96]	4.95 [2.12]
Migrant has less than high school education	0.62	0.61
Migrant has high school education or more	0.38	0.39
Observations	1298	2208

Notes: Baseline survey samples are observations with non-missing values for all experiment questions and completed recipient survey. Sample size varies slightly with variable: 1264 for age; 1295 for years in US; 1290 for education variables. ACS sample is the IPUMS three year 2008–2010 ACS sample restricted to individuals 18–65 in the Washington, DC metro area (as defined by the ACS, includes MD and VA suburbs). Standard deviation in brackets for continuous variables.

**Appendix Table 2:** Relationship of migrant to recipient.

The recipient is the migrant's…	Percent
Spouse	2.87
Parent	13.09
Daughter/son	15.18
Sibling	28.66
Grandparent	0.39
Grandchild	0.70
Niece/nephew	14.87
Cousin	7.51
Aunt/uncle	3.49
Parent-in-law	1.47
Daughter/son-in-law	0.39
Sister/brother-in-law	4.03
Friend	2.25
Other	5.11

**Appendix Table 3:** Variables for subgroup analysis.

Variable	Expected correlation with remittance costs/benefits	Justification	Subgroup definition
Migrant years in the United States:	Negative	A migrant's reputation at home is important for migrants who wish to return, and the probability of return may decline with length of time in the United States. With time it is also more likely that the migrant has paid off any debts related to initial migration costs.	The median number of years in the United States is 10.
Migrant has a child 22 or under in El Salvador	Positive	Migrants may have left children in the care of the recipient, and the possibility of child care that does not meet the migrant's preferences could be a powerful tool.	34% of migrants have a son or daughter aged 22 or under in El Salvador.
Migrant and recipient are closely related	Positive	This is defined as spouses or parent and child. Migrants may have entrusted recipients with the care of things that are important to them, and positive relationships with the recipients may be valuable to the migrants. Social norms may also be stronger in this group.	31% of migrants and recipients are closely related.
Migrant communicates with recipient household weekly	Positive	Frequent communication is a sign that migrants value their relationships with recipients.	71% of migrants report communicating weekly with the recipient household.
Remittances sent by migrant to recipient household	Positive	Because remittance relationships where recipients induce migrants to send money result in higher payments, higher remittances may indicate high costs.	The median annual remittance total to the recipient household reported by the migrant is $1,800.

**Appendix Table 4:** Balance tests by subgroup: migrant experiment.

	P-value for difference of means: choice not revealed and choice revealed									
	
	Years in the United States		Migrant has child 22 or under in El Salvador		Recipient is close relative of migrant		Migrant communicates with recipient household weekly		Migrant's annual remittances to recipient household	
					
	Above sample median	Below sample median	No	Yes	No	Yes	No	Yes	Below sample median	Above sample median
*Baseline variables from US survey*										
Migrant is female	0.206	0.588	0.228	0.587	0.155	0.686	0.481	0.335	0.694	0.078
Migrant age	0.674	0.736	0.696	0.684	0.413	0.371	0.069	0.170	0.644	0.534
Migrant can read and write	0.925	0.026	0.406	0.169	0.277	0.268	0.938	0.131	0.630	0.029
Migrant's years of education	0.797	0.869	0.787	0.386	0.688	0.452	0.744	0.952	0.121	0.246
Migrant is married	0.045	0.881	0.047	0.624	0.260	0.407	0.587	0.148	0.388	0.222
Migrant lives with spouse	0.418	0.329	0.052	0.025	0.651	0.886	0.168	0.340	0.133	0.176
Migrant's total number of children	0.589	0.893	0.714	0.105	0.322	0.432	0.624	0.560	0.257	0.339
Migrant's number of children in El Salvador	0.825	0.288	0.809	0.513	0.733	0.600	0.509	0.070	0.584	0.333
Migrant's number of children in US	0.433	0.187	0.793	0.205	0.190	0.528	0.886	0.195	0.390	0.009
Migrant's hh size in US	0.308	0.646	0.990	0.316	0.768	0.912	0.796	0.710	0.560	0.896
Migrant has worked in last 12 months	0.139	0.291	0.994	0.582	0.738	0.717	0.484	0.584	0.904	0.647
Migrant in lowest income bracket	0.442	0.537	0.460	0.631	0.320	0.230	0.811	0.936	0.815	0.971
*Baseline variables from recipient survey*										
Recipient is target student	0.775	0.514	0.581	0.208	0.851	0.358	0.631	0.643	0.989	0.899
Recipient is student's guardian	0.266	0.358	0.550	0.071	0.279	0.150	0.841	0.099	0.312	0.334
Recipient is female	0.399	0.579	0.976	0.045	0.307	0.543	0.654	0.177	0.831	0.260
Recipient age	0.147	0.120	0.373	0.020	0.102	0.070	0.620	0.041	0.385	0.071
Recipient is married	0.287	0.296	0.315	0.195	0.499	0.477	0.749	0.857	0.752	0.712
Recipient's years of education	0.368	0.477	0.807	0.014	0.285	0.349	0.942	0.237	0.956	0.194
Recipient lives in urban area	0.135	0.374	0.966	0.414	0.581	0.881	0.361	0.972	0.393	0.787
Recipient's hh size	0.115	0.503	0.026	0.718	0.089	0.459	0.467	0.136	0.531	0.109
Annual remittances received from migrant ($)	0.620	0.712	0.892	0.940	0.246	0.297	0.168	0.503	0.102	0.692

Notes: Samples are observations with non-missing values for the experiment questions and completed recipient survey. Sample size for each comparison of means varies slightly by missing values for each variable. Other notes on variable construction are as in Table 1. P-values come from a regression of each variable on treatment, with standard errors adjusted for heteroskedasticity.

**Appendix Table 5:** Impact of monitoring and communication treatments on recipient allocation decision: by information quality measure.

	(1)		(2)	(3)		(4)
	
	Dependent variable: Total migrant–recipient difference						
	
	Migrant correctly reports student GPA			Migrant correctly reports student mode of transport to school		
		
	No		Yes	No		Yes
Recipient choice revealed to migrant	1.222 [8.417]		0.572 [22.75]	0.0117 [9.768]		4.654 [11.81]
Migrant preference revealed to recipient	−17.78** [8.515]		2.059 [20.20]	−22.71** [10.25]		−10.94 [12.04]
Choice revealed * preference revealed	2.028 [12.38]		5.498 [31.54]	−2.648 [14.81]		12.54 [16.99]
*P-values for equality of treatment effects*:						
Monitoring treatment		0.976			0.761	
Communication treatment		0.311			0.454	
Monitoring * communication		0.908			0.499	
Observations	787		254	633		474
R-squared	0.157		0.397	0.228		0.243
Mean in recipient choice not revealed, migrant preference not revealed	146.3		129.2	150.0		135.0

Notes: Robust standard errors are in brackets. Samples are observations with non-missing values for experiment questions, completed recipient survey and non-missing values for variables used for division into sub-samples. Responses to GPA and transport questions were only recorded if student was reported to be in school. Migrants were asked to report the student's GPA within a 2 point (out of 10) range, while recipients reported an exact number. The migrant is said of have correctly reported the GPA if the recipient's response was within the range the migrant indicated. All regressions include stratification group fixed effects: dummy variables for the groups of survey numbers within which randomization was stratified and treatment status in the migrant experiment.

** p < 0.05,

**Appendix Table 6:** Impact of monitoring and communication treatments on recipient allocation decision: amounts allocated by recipient.

	(1)	(2)	(3)	(4)
	
	Dependent variable: amount allocated by recipient to…			
	
	Restaurant spending	Education spending	Daily expenses spending	Health spending
Recipient choice revealed to migrant	−1.189 [1.413]	−10.36 [7.722]	16.83*** [5.716]	−5.286 [5.242]
Migrant preference revealed to recipient	0.628 [1.517]	−1.591 [7.546]	2.717 [5.447]	−1.754 [5.214]
Choice revealed * preference revealed	0.616 [2.216]	4.733 [10.70]	−14.15* [7.802]	8.799 [7.344]
Observations	1298	1298	1298	1298
R-squared	0.129	0.117	0.105	0.107
Mean in recipient choice not revealed, migrant preference not revealed	17.99	116.2	78.33	77.94

Notes: Robust standard errors are in brackets. Samples are observations with non-missing values for all experiment questions and completed recipient survey. Dependent variables are the raw amounts allocated by recipient to different spending categories. All regressions include stratification group fixed effects: dummy variables for the groups of survey numbers within which randomization was stratified and treatment status in the migrant experiment.

*** p < 0.01,

* p < 0.1.

## Appendix E. Description of pilot studies

Prior to implementation of the experiment a small pilot was conducted to test the clarity of the questions. The pilot was conducted as part of a pilot test of the baseline survey and consisted of approximately 20 participants. The pilot was also used to calibrate the amounts that were used in the experimental questions and the categories that were offered in the recipient experiment. The pilot also tested the format of the migrant experiment: whether or not migrants were required to split their allocation in $100 increments.
