# Supplementary material for: Don't tell on me: Experimental evidence of asymmetric information in transnational households
Source: J Dev Econ. Author manuscript; Available in PMC 2016 Mar 1. (PMC4280859; doi:10.1016/j.jdeveco.2014.11.001)
Supplement: Supplement [file NIHMS646793-supplement-Supplement.zip › Don't Tell on Me Replication Files/readme.pdf]

These files recreate the analysis in the main text and appendices of "Don't Tell on Me: Experimental Evidence of Asymmetric Information in Transnational Households."

By: Kate Ambler, IPFRI

To run these files do the following:

1. Set your stata directory to this folder.
2. To construct the variables used in our analysis from the raw survey data run the do file "variable.prep.do". The data input is merged_surveys.dta and the output will be analysis_data.dta.
3. To recreate the analyses run the file "analysis.do" which will run all the analysis do files contained in the analysis folder. All output will be sent to the reg_output folder.
4. All analysis files use the dataset analysis_data.dta with the following exception:
    a. Appendix Table 1 uses the 3 year (2008 – 2010) ACS publicly available from IPUMS. Interested users can download the data from the IPUMS website. When requesting the data extract, request only observations from the Washington, DC metro area.
